# SETD2 loss of function is a recurrent event in advanced‐phase chronic myeloid leukemia and contributes to genomic instability: SETD2 loss in Chronic Myeloid Leukemia

**DOI:** 10.1002/ctm2.70163

**Published:** 2025-04-24

**Authors:** Manuela Mancini, Sara De Santis, Cecilia Monaldi, Fausto Castagnetti, Miriam Iezza, Alessandra Iurlo, Daniele Cattaneo, Sara Galimberti, Marco Cerrano, Isabella Capodanno, Massimiliano Bonifacio, Maura Rossi, Claudio Agostinelli, Manja Meggendorfer, Torsten Haferlach, Michele Cavo, Gabriele Gugliotta, Simona Soverini

**Affiliations:** ^1^ IRCCS Azienda Ospedaliero‐Universitaria di Bologna Istituto di Ematologia “Seràgnoli” Bologna Italy; ^2^ Department of Medical and Surgical Sciences University of Bologna Bologna Italy; ^3^ Hematology Division Foundation IRCCS Ca' Granda Ospedale Maggiore Policlinico Milano Italy; ^4^ Department of Oncology and Hemato‐Oncology University of Milan Milano Italy; ^5^ Clinical and Experimental Medicine Hematology University of Pisa Pisa Italy; ^6^ Azienda Ospedaliera Citta' Della Salute E Della Scienza Di Torino Torino Italy; ^7^ SOC Ematologia Azienda USL‐IRCSS di Reggio Emilia Reggio Emilia Italy; ^8^ Section of Hematology Department of Medicine Azienda Ospedaliera Universitaria Integrata di Verona Verona Italy; ^9^ Haematopathology Unit IRCCS Azienda Ospedaliero‐Universitaria di Bologna Bologna Italy; ^10^ MLL Munich Leukemia Laboratory Munich Germany

**Keywords:** Aurora kinase A, genomic instability, H3K36me3, MDM2, SETD2

## Abstract

**Abstract:**

The *SETD2* tumour suppressor encodes a histone methyltransferase that specifically trimethylates histone H3 on lysine 36 (H3K36me3), a key histone mark implicated in the maintenance of genomic integrity among other functions. We found that SETD2 protein deficiency, mirrored by H3K36me3 deficiency, is a nearly universal event in advanced‐phase chronic myeloid leukemia (CML) patients. Similarly, K562 and KCL22 cell lines exhibited markedly reduced or undetectable SETD2/H3K36me3 levels, respectively. This resulted from altered SETD2 protein turnover rather than mutations or transcriptional downregulation, and proteasome inhibition led to the accumulation of hyper‐ubiquitinated SETD2 and to H3K36me3 rescue suggesting that a functional SETD2 protein is produced but abnormally degraded. We demonstrated that phosphorylation by Aurora‐A kinase and ubiquitination by MDM2 plays a key role in the proteasome‐mediated degradation of SETD2. Moreover, we found that SETD2 and H3K36me3 loss impinges on the activation and proficiency of homologous recombination and mismatch repair. Finally, we showed that proteasome and Aurora‐A kinase inhibitors, acting via SETD2/H3K36me3 rescue, are effective in inducing apoptosis and reducing clonogenic growth in cell lines and primary cells from advanced‐phase patients. Taken together, our results point to SETD2/H3K36me3 deficiency as a mechanism, already identified by our group in systemic mastocytosis, that is reversible, druggable, and BCR::ABL1‐independent, able to cooperate with BCR::ABL1 in driving genetic instability in CML.

**Key Points:**

- Virtually all CML patients in blast crisis display SETD2 loss of function.
- SETD2 loss seems to be accomplished at the posttranslational level rather than being the result of genetic/genomic hits or transcriptional repression.
- Phosphorylation by Aurora kinase A and ubiquitination by MDM2 contribute to SETD2 proteasome‐mediated degradation in blast crisis CML patients.
- Loss of SETD2 results in increased DNA damage.

## Graphical abstract

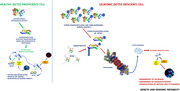

## 1 INTRODUCTION

The driver event in the pathogenesis of chronic myeloid leukemia (CML) is the t(9;22) chromosomal translocation, resulting in a *BCR::ABL1* fusion gene that encodes a protein with constitutively active tyrosine kinase activity.^1^ The development of ATP‐competitive and allosteric tyrosine kinase inhibitors (TKIs) has profoundly impacted the prognosis of CML patients, so that those who achieve optimal response to therapy have a life expectancy almost superimposable to that of age‐ and sex‐matched healthy individuals,^2^ and a not negligible proportion may even discontinue TKI treatment.^3^ CML has traditionally been characterized by a triphasic clinical course. Almost all patients are diagnosed in the initial chronic phase (CP), which displays a rather indolent clinical course but may later progress to the accelerated phase (AP; now redefined ‘high‐risk CP CML by the 5th edition of the World Health Organization [WHO] classification) and ultimately to blast crisis (BC).^4^, ^5^ Disease evolution into BC, which resembles acute leukemia, is known to be sustained by an ever‐increasing genetic instability fostering the accumulation of additional genetic ‘hits’ that progressively reduce oncogenic addiction of CML cells to BCR::ABL1.^4^ Although, in the TKI era, the proportion of CP patients progressing to BC has greatly decreased, those who do have a poor prognosis due to reduced long‐term efficacy of TKIs and to the lack of effective therapeutic alternatives.^6^ Disease progression is thought to be a multifactorial and probably multistep phenomenon, whose underlying mechanisms are not fully understood.

In recent years, the deregulation of epigenetic modifiers has turned out to be a recurrent event in cancer evolution. The SETD2 tumour suppressor gene encodes a histone methyltransferase that catalyzes the trimethylation of lysine 36 on histone H3 (H3K36me3),^7^ a key histone mark implicated in transcriptional elongation, alternative splicing, and DNA repair.^8^, ^9^, ^10^, ^11^, ^12^, ^13^, ^14^ Lately, SETD2 has also been shown to methylate α‐tubulin during mitosis, ensuring the fidelity of chromosomal segregation and cytokinesis.^15^ Loss of function of SETD2, mainly via biallelic inactivating (frameshift or nonsense) mutations or deletion of one allele and mutation of the remaining allele, is known to occur in many solid tumours^16^, ^17^ and in acute myeloid and lymphoid leukemias.^18^, ^19^, ^20^ In all cases, it correlates with aggressive and relapsed disease.^19^, ^21^, ^22^, ^23^, ^24^ Furthermore, our group has recently reported that SETD2 loss of function is also frequent in advanced systemic mastocytosis, where it mainly occurs at the post‐translational rather than at the genomic level, via hyper‐ubiquitination and proteasomal degradation. As such, it is potentially reversible.^25^

Based on these premises, and in an attempt to identify potentially druggable players of CML progression, we investigated whether advanced‐phase CML, which shares similarities with acute leukemias such as a high degree of genetic instability, might also exhibit loss of function of the SETD2 gene.

## 2 MATERIALS AND METHODS

### 2.1 Cell lines and patient samples

K562, LAMA84 and KCL22 BCR::ABL1‐positive cell lines were maintained in RPMI 1640 medium (Lonza Group LTD) supplemented with 10% fetal calf serum (FCS, Gibco, Thermo Fisher Scientific), 1% L‐glutamine and antibiotics in 5% CO_2_ and fully humidified atmosphere at 37°C.

These three cell lines were selected because they were found by Western blotting to exhibit different SETD2 protein levels. In detail: LAMA84 cells express high levels of SETD2, thus were used to transiently silence the SETD2 gene; in contrast, KCL22 cells show no evidence of the SETD2 protein, thus were used to force SETD2 re‐expression by nucleofection of a SETD2 plasmid. K562 express intermediate levels of the SETD2 protein;

Primary sample collection and patients’ characteristics are detailed in the supplementary information.

### 2.2 *SETD2* sequencing

*SETD2* coding and promoter sequences were screened in genomic DNA obtained from cell lines and patient samples using high throughput sequencing as previously described.^26^

### 2.3 Quantitative reverse transcription (RT)‐polymerase chain reaction (PCR) for *SETD2* expression

*SETD2* expression was assessed on an ABI 7900HT system (Thermo Fisher Scientific) using pre‐designed TaqMan gene expression assays (Thermo Fisher Scientific) for *SETD2* (Hs01014784_m1) and *GUSB* (Hs99999908_m1) endogenous control as previously described.^26^ SETD2 mRNA levels were quantified using the Comparative Ct method, using a pool of 10 HD of various ages as a calibrator.

### 2.4 RNA interference

RNA interference experiments were performed as described in the Supporting Information.

### 2.5 Cell transfection

SETD2 forced expression in KCL22 cells was performed by nucleofection according to the manufacturer's instructions. Briefly, KCL22 cells were harvested, washed, and resuspended in a transfection reagent (Lonza). Subsequently, 80 µL of the cell suspension containing 1 × 10^6^ cells was mixed with 2 µg of a GFP‐tagged *SETD2* construct (Origene) and electroporated in a .2 cm cuvette using the Nucleofector 2b device (Lonza) with the Lonza Cell Line Nucleofection Kit V according to the Lonza Amaxa 4D‐Nucleofector Protocol.

GFP‐transfected cells were checked for GFP expression 24 and 48 h after nucleofection by flow cytometry. Briefly, cells (5 × 10^5^ cells) were washed once in phosphate‐buffered saline (PBS) and resuspended in 200 µL PBS and acquired by using a CytoFLEX analytical flow cytometer (Beckman Coulter) to assess GFP positivity.

SETD2 re‐expression and reactivation were confirmed by Western blotting (Figure S2B), and forced expression of SETD2 induced a significant increase in doubling time associated with an accumulation of cells at the G1/S and G2/M checkpoints (Figure S2C,D).

### 2.6 Western blotting and co‐immunoprecipitation/immunoblotting

Western blotting (WB) and co‐immunoprecipitation/immunoblotting (co‐IP) were performed as described in the supplementary information using the following antibodies: anti‐SETD2 (Genetex), anti‐H3K36me3, anti‐ubiquitin, anti‐Aurora‐A kinase, anti‐phospho‐Aurora‐A kinase(T288), anti‐phospho‐H2AX S(139), anti‐Rad51, anti‐ATM, anti‐phospho‐ATM S(1981), anti‐p95, anti‐phospho‐p95 S(343), anti‐CtIP (Cell Signaling Technology). Beta‐actin (Santa Cruz Biotechnology) or beta‐tubulin (Cell Signaling Technology) were used as loading controls.

### 2.7 Immunofluorescence

Cells set on poly‐L‐lysine‐coated glass slides were fixed with 3.7% paraformaldehyde in PBS for 10 min at 37°C, washed three times with .1 M glycine in PBS, permeabilized in 70% ice‐cold ethanol for 2 min at −20°C and incubated overnight at 4°C with primary antibodies as described in the supplementary information. Images were acquired using an Axiovert 40 CFL microscope (Carl Zeiss S.p.A.). Images were acquired with a 100× objective.

### 2.8 Drug treatments

All drugs used for cytotoxicity and functional experiments are detailed in the supplementary information.

### 2.9 Statistical analysis

Data are presented as mean ± standard deviation (SD) and were analyzed for statistical significance by Student *t*‐test (GraphPad Prism Software). The *t*‐test inferential statistic was chosen as the most suitable method to determine if there is a significant difference between the means of two groups compared. The two groups compared were indicated in each figure legend. A *p* value < .05 was considered statistically significant.

## 3 RESULTS

### 3.1 H3K36me3 is reduced or lost in the great majority of advanced‐phase CML patients and in some CML cell lines

**FIGURE 1:**
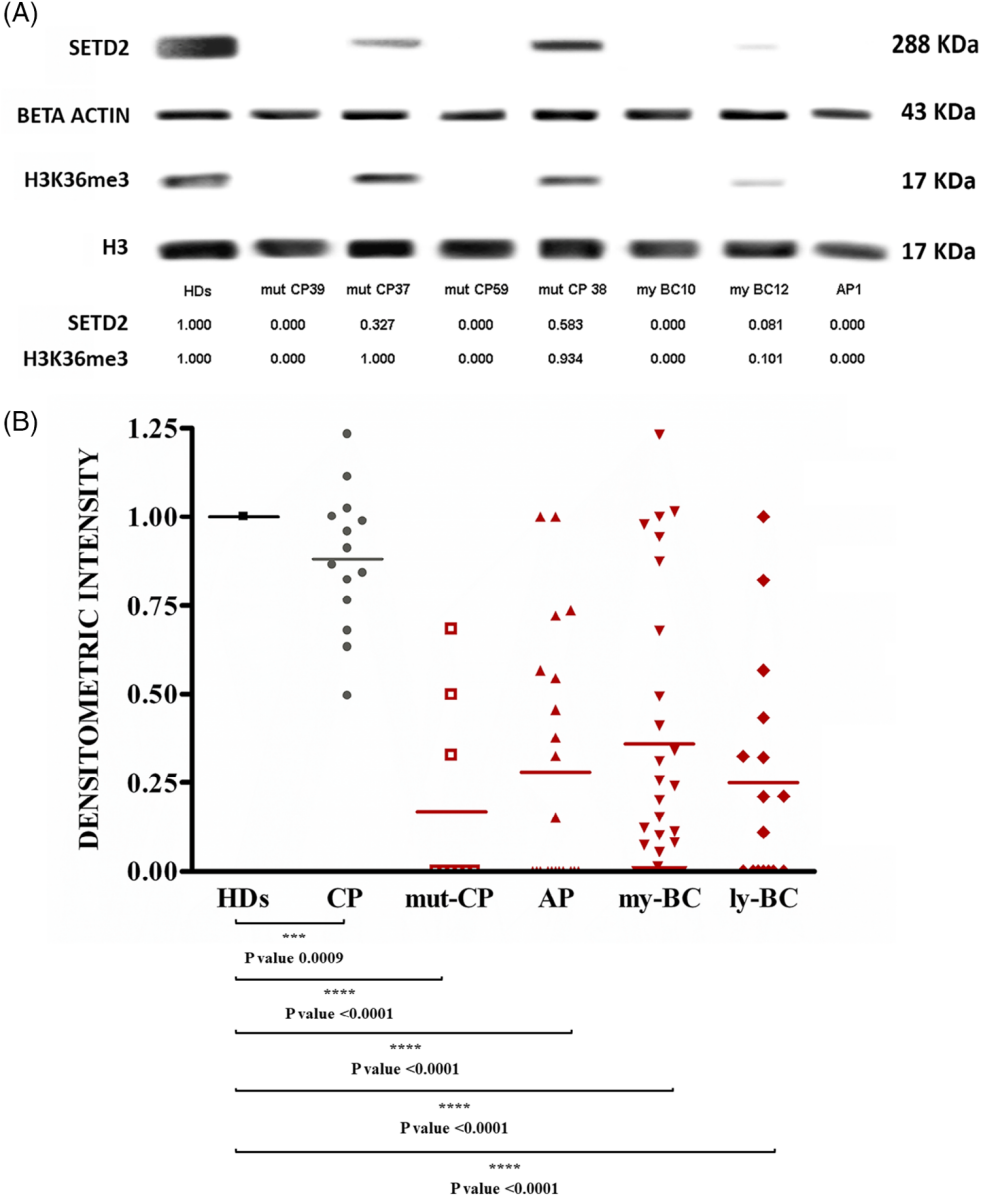
SETD2 deficiency in advanced phases of CML. (A) Representative Western blot results for SETD2 protein and H3K36me3 levels in CML patients as compared with a pool of healthy donors (HDs). One of three independent experiments is shown. (B) Scatter plot of SETD2 levels estimated by densitometric analysis of Western blots. The median is indicated for each group. SETD2 signal intensities in single blots obtained from three individual experiments were normalized to those of beta‐actin and averaged. Normalized SETD2 levels calculated in CML patients were then expressed in comparison to normalized SETD2 levels detected in a pool of HDs, conventionally set to 1. The Student's *t*‐test was used to compare the means between two groups (CP vs. HDs; mut‐CP vs. HDs; AP vs. HDs; my‐BC vs. HDs and finally ly‐BC vs. HDs). ***p < .0009; ****p < .0001.

Since SETD2 is the only methyltransferase that can trimethylate histone H3 on Lysine 36 in humans,^7^ we assessed H3K36me3 levels by WB as a surrogate marker of SETD2 loss of function. WB with an antibody against the N‐terminal region of SETD2 was also performed in parallel. With this approach, we screened a total of 84 advanced‐phase CML patients, including patients in AP (according to the 4th edition of the WHO classification; *n* = 21), myeloid BC (*n* = 38), lymphoid BC (*n* = 16) and patients with multi‐TKI‐resistant CP harbouring BCR::ABL1 kinase domain mutations (mut‐CP; *n* = 9). Samples collected at diagnosis from CP patients (*n* = 9) who later achieved optimal response to therapy were also studied for comparison. Reduced or null H3K36me3 (as compared with a pool of healthy donors [HD]) was detected in the great majority of patients with advanced‐phase CML (74/84, 88%; 19/21 AP, 32/38 my‐BC, 14/16 ly‐BC, 9/9 mut‐CP). In contrast, CP patients at diagnosis showed H3K36me3 levels superimposable to those of HD (Figure 1). No evidence of abnormal SETD2 protein isoforms was detected, but patients with no H3K36me3 also had no detectable SETD2 protein and patients with reduced H3K36me3 had similarly reduced levels of full‐length SETD2 protein as assessed by densitometric analysis of WB (Spearman *R* = .95, *p* < .001). Since the low percentage of blasts in CP patients might be the reason why SETD2 expression and activity were found to be comparable to those of the healthy population, we next explored the expression levels of SETD2 in the CD34+ subpopulation (representative of leukemic BCR::ABL1‐positive progenitors) of 14 patients in CP (who displayed a percentage of CD34+ cells in the BM ranging between .5 and 3%) and 3 patients in advanced‐phase (who displayed a percentage of CD34+ cells in the BM ranging between 20% and 30%). As shown in Figure 1S, the CD34+ subpopulation of CP and advanced‐phase patients shows superimposable SETD2 expression levels.

We next wondered whether the same could be observed in CML cell lines. Hence, K562, LAMA84, and KCL22 cells were similarly screened by WB. SETD2 and H3K36me3 levels were found to be null in KCL22, low in K562 and high in LAMA84 (Figure 3SA).

### 3.2 SETD2 deficiency in advanced‐phase CML occurs at the posttranslational level

**FIGURE 2:**
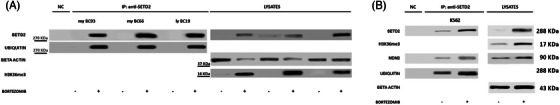
Effects of proteasomal inhibition on SETD2 expression and function. Co‐immunoprecipitation assays show that (A) in samples from SETD2‐deficient patients, bortezomib treatment rescued a hyper‐ubiquitinated SETD2 protein; (B) in K562 cells showing low basal SETD2 levels, bortezomib treatment increased SETD2 protein levels. SETD2 appears to be ubiquitinated and to bind MDM2 in the same context. A negative control (NC) obtained by immunoprecipitation of protein lysates using a resin conjugated with an anti‐IgG1 antibody was loaded for each immunoprecipitation experiment. One of three independent experiments is shown in all panels.

To investigate the mechanisms underlying the observed SETD2 loss, the whole coding region and the promoter of the *SETD2* gene were screened for mutations by high throughput sequencing. Point mutations were observed in only 2/84 patients: one patient had a G1231E mutation (previously reported in T‐cell acute lymphoblastic leukemia^27^ and breast cancer^28^) and another patient had an R493Q (previously reported in an astrocytoma).^29^ We next assessed *SETD2* mRNA levels by RT‐qPCR, but they appeared not to be significantly lower in patients with no or low SETD2 expression as compared with HD (Figure 2S), which led us to exclude the occurrence of gene deletions, haploinsufficiency or promoter hyper‐methylation. Thus, SETD2 deficiency in advanced‐phase CML had to be ascribed to a mechanism acting at the translational or post‐translational level. Incubation with an inhibitor of proteasome‐mediated degradation (bortezomib) for 24 h rescued the expression of a functional SETD2 protein, as shown by WB for SETD2 and H3K36me3 (Figure 2A). Immunoprecipitation (IP) with an anti‐SETD2 antibody after bortezomib treatment showed that blockage of proteasomal degradation results in the accumulation of hyper‐ubiquitinated SETD2 (Figure 2A). The same was observed in the KCL22 and K562 cell lines: no mutations or reduced transcript levels as compared with LAMA84 cells were detected, but proteasome inhibition increased both SETD2 protein expression and H3K36me3 levels (Figure 2B). Taken together, these findings indicate that in advanced‐phase CML a functional SETD2 protein is produced, but it undergoes hyper‐ubiquitination and rapid proteasome mediated‐degradation which ultimately results in H3K36me3 deficiency.

### 3.3 Phosphorylation by Aurora‐A kinase and ubiquitination by MDM2 contribute to proteasome‐mediated degradation of SETD2 in advanced‐phase CML

**FIGURE 3:**
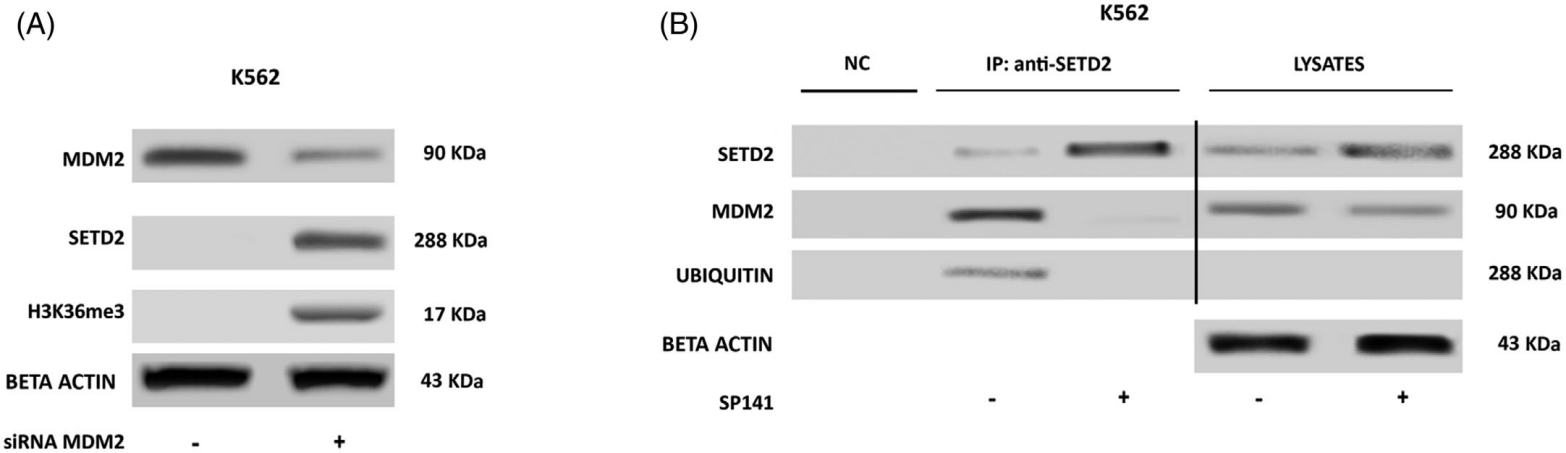
Effects of MDM2 inhibition on SETD2 expression and function. Western blotting and co‐immunoprecipitation assays show that (A) silencing or (B) inhibiting MDM2 by siRNA (for 72 h) or by SP141 (5 µM for 24 h), respectively, rescued SETD2/H3K36me3 expression in K562 cells. A negative control (NC) obtained by the immunoprecipitation of protein lysates using a resin conjugated with an anti‐IgG1 antibody was loaded for each immunoprecipitation experiment. One of three independent experiments is shown in all panels.

The observation that in advanced‐phase CML loss of function of SETD2 is most frequently nongenetic/nongenomic, hence reversible, prompted us to investigate the underlying mechanisms. First of all, we set to assess whether SETD2 loss of function is dependent upon BCR::ABL1 kinase activity. K562 and KCL22 cells were treated with imatinib 1 µM and nilotinib 100 nM for 24 and 48 h and SETD2 expression and activity were evaluated by WB. Neither imatinib nor nilotinib treatment‐induced changes in SETD2 expression in any cell line (data not shown). Having excluded a BCR::ABL1 kinase‐dependent mechanism, we aimed to identify other key (and possibly druggable) players. Several lines of evidence in normal and neoplastic cell line models have recently concurred to indicate that SETD2 can bind and contribute to p53 activation.^30^, ^31^ We thus reasoned that should the same occur in advanced‐phase CML, then MDM2, which is physiologically responsible for p53 ubiquitination,^32^ might also target SETD2. To explore this hypothesis, we first performed co‐IP assays. In K562 (SETD2/H3K36me3‐low) cells we found that MDM2 co‐immunoprecipitates with hyper‐ubiquitinated SETD2, and this is enhanced after proteasome inhibition (Figure 2B). MDM2 knock‐down significantly increased SETD2 and H3K36me3 levels (Figure 3A), and this was phenocopied by pharmacologic targeting of MDM2 with SP‐141,^33^, ^34^, ^35^ a highly specific small molecule that inhibits MDM2 by promoting MDM2 autoubiquitination and proteasomal degradation (Figure 3B).

**FIGURE 4:**
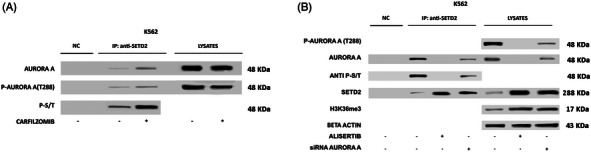
Aurora‐A kinase interacts with SETD2 and its inhibition affects SETD2 expression and function. Western blotting and co‐immunoprecipitation assays show that (A) proteasomal inhibition by carfilzomib (5 nM for 24 h) rescuing SETD2 expression reveals its interaction with Aurora‐A kinase; (B) both siRNA‐mediated silencing of Aurora‐A for 72 h and Aurora‐A inhibition by alisertib (100 nM for 24 h) rescued SETD2/H3K36me3 expression in K562 cells and resulted in SETD2 release from Aurora‐A kinase and SETD2 dephosphorylation on Ser/Thr residues. A negative control (NC) obtained by the immunoprecipitation of protein lysates using a resin conjugated with an anti‐IgG1 antibody was loaded for each immunoprecipitation experiment. One of three independent experiments is shown in all panels.

To identify additional interactors that might be implicated in SETD2 loss of function, we next interrogated protein interaction databases. In both IntAct^36^ and BioGRID,^37^ we spotted Aurora‐A kinase among SETD2‐interacting proteins. This intrigued us since we had previously shown that Aurora‐A kinase is overexpressed and hyperactivated in TKI‐resistant CML.^38^, ^39^ In particular, we had observed that selection of resistant K562 (K562‐R) cells by exposure to increasing doses of imatinib resulted in marked upregulation and activation of Aurora‐A kinase as compared with parental K562.^38^ We thus investigated SETD2 and H3K36me3 levels in K562‐R cells, finding that, in contrast to parental K562, both SETD2 expression and H3K36me3 are completely abrogated. We thus hypothesized that aberrant expression and activation of Aurora‐A kinase may supply the phosphorylation signals that trigger SETD2 ubiquitination in advanced‐phase CML. To investigate this issue, we again performed co‐IP assays. We found that Thr288 phosphorylated (active)‐Aurora‐A co‐immunoprecipitates with SETD2 in K562 cells, and SETD2 band intensity increased after proteasome‐mediated degradation was blocked with carfilzomib (Figure 4A). Moreover, SETD2 IP labelled with an anti‐phospho Ser‐Thr antibody showed that Aurora‐A kinase interaction with SETD2 results in the phosphorylation of SETD2. Knock‐down of Aurora‐A in K562 cells markedly increased SETD2 expression and H3K36me3 (Figure 4B). Similarly, pharmacologic treatment with alisertib, a highly potent and selective inhibitor of Aurora‐A, disrupted the Aurora‐A‐SETD2 complex, abrogated SETD2 Ser/Thr phosphorylation and resulted in a marked increase of total SETD2 and H3K36me3 levels (Figure 4B).

### 3.4 SETD2/H3K36me3 deficiency contributes to genetic instability

It is known from the literature that H3K36me3 plays a key role in maintaining genomic integrity by modulating several DNA repair pathways, including homologous recombination (HR) and mismatch repair (MMR).^31^, ^40^, ^41^

**FIGURE 5:**
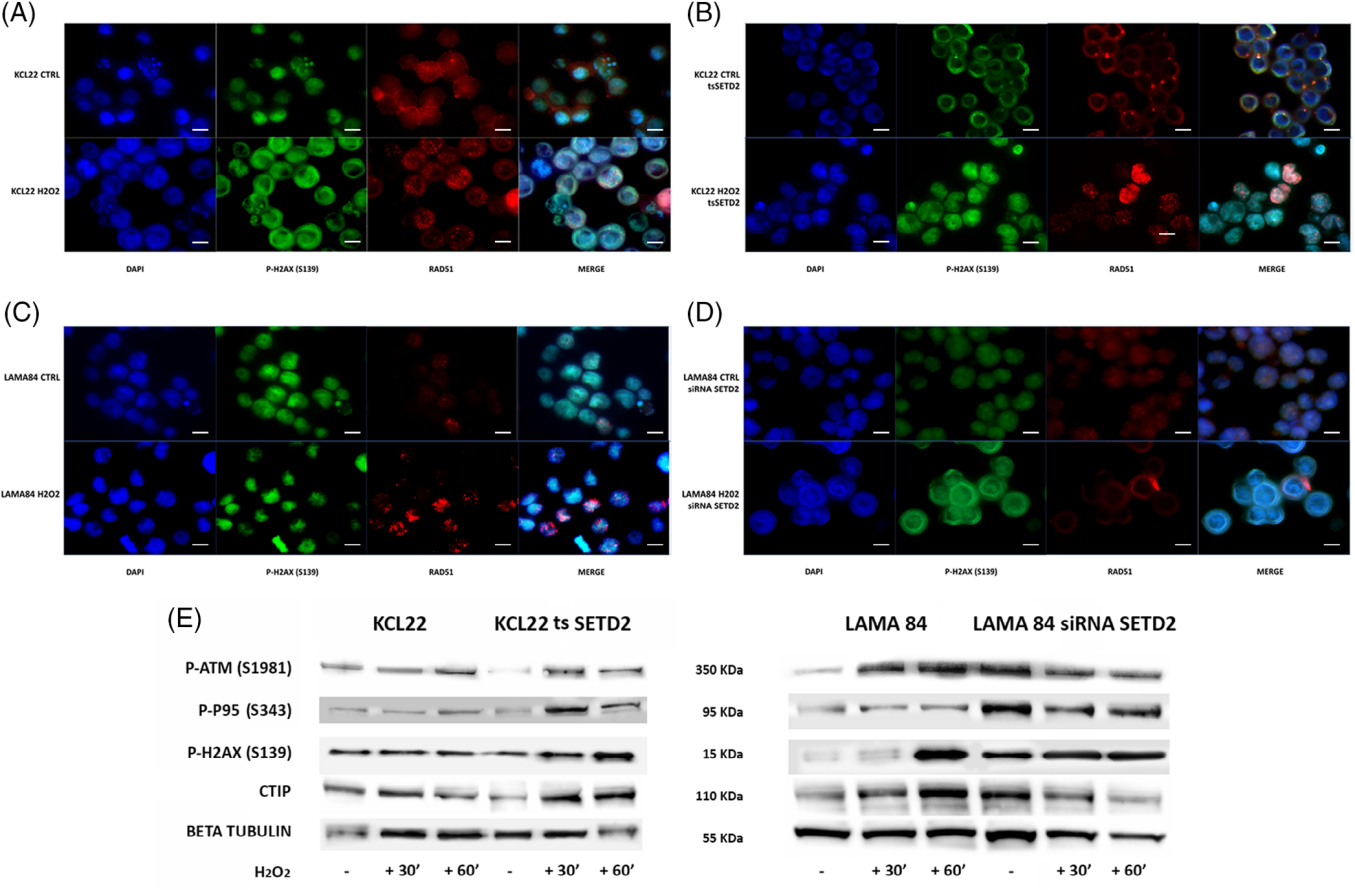
Effects of SETD2 expression on HR repair activation. (A–D) Immunofluorescence analysis of phosphorylated histone 2A.X (P‐H2AX(S139), green) and Rad51 (red) in SETD2‐deficient and SETD2‐proficient cells after sub‐lethal DNA damage induction by hydrogen peroxide exposure. Staining with DAPI (4′,6‐diamidino‐2‐phenylindole) indicates the nuclear localization of P‐H2AX(S139) and Rad51. (A) KCL22 (SETD2‐deficient) cells; (B) KCL22 tsSETD2 cells; (C) LAMA84 (SETD2‐proficient) cells; (D) LAMA84 silenced for SETD2. Scale bar: 100 µM. (E) Western blotting showed that SETD2 loss impairs ATM signalling pathway activation; in contrast, SETD2 forced expression restores the ability to induce ATM phosphorylation and subsequent p95 and H2AX activation, associated with an increase in CtIP expression.

To investigate whether SETD2 and H3K36me3 deficiency impair the activation of HR repair in advanced‐phase CML, LAMA84 (SETD2/H3K36me3‐proficient) cells before and after SETD2 silencing (Figure 3SB) and KCL22 (SETD2/H3K36me3‐deficient) cells before and after SETD2 transfection (Figure 3SC,D) were studied by immunofluorescence to assess phosphorylated H2AX (a DNA double‐strand break [DSB] marker) and Rad51 (a marker of ongoing HR) foci in steady‐state conditions and after hydrogen peroxide (H_2_O_2_) exposure (1 mM for 60 min) or chronic UV exposure. KCL22 cells were unable to activate HR repair after the induction of DSBs, as demonstrated by the widespread distribution of the two proteins, and were unable to colocalize in discrete foci (Figure 5A). Superimposable results were obtained in SETD2‐depleted cells (LAMA84 siSETD2; Figure 5D).

In contrast, cells proficient for SETD2 (KCL22 tsSETD2 and LAMA84) displayed the ability to activate HR after H_2_O_2_ exposure by inducing the formation of DNA repair foci mediated by phosphorylated H2AX and RAD51 co‐localization (Figure 5B,C).

To assess whether SETD2 and H3K36me3 loss impairs the ability to activate the ATM‐dependent repair pathway, we used WB to check the effects of H_2_O_2_ on ATM phosphorylation status and activation of its downstream pathway in SETD2‐deficient and SETD2‐proficient cells. We found that failed ATM activation in KCL22 cells and in LAMA84 siSETD2 impaired p95 and H2AX phosphorylation, and reduced CtIP expression (CtIP is a key regulator of DNA DSB since it promotes the resection of 5′ strands to generate 3′ single‐stranded intermediates that are necessary for HR. This activity requires CDK‐dependent association with p95 after phosphorylation by ATM^42^). As a confirmation, SETD2 forced re‐expression in KCL22 cells was able to restore DNA damage response as demonstrated by ATM and H2AX phosphorylation and CtIP expression after H_2_O_2_ exposure (Figure 5E). ATM, p95, and RAD51 expression were not significantly affected by DNA damage induction in our in vitro models (Figure 4S).

Finally, we used immunofluorescence to assess the activation of HR repair after chronic UV exposure in SETD2‐proficient (KCL22 tsSETD2 and LAMA84) versus SETD2‐deficient cells (KCL22 and LAMA84 siSETD2). Our results confirmed the inability of SETD2‐deficient cells to activate HR, in contrast to SETD2‐proficient cells who displayed H2AX phosphorylation and RAD51 recruitment on DNA damage foci (Figure 5S). Taken together, these observations support the role of SETD2 in HR repair in CML.

**FIGURE 6:**
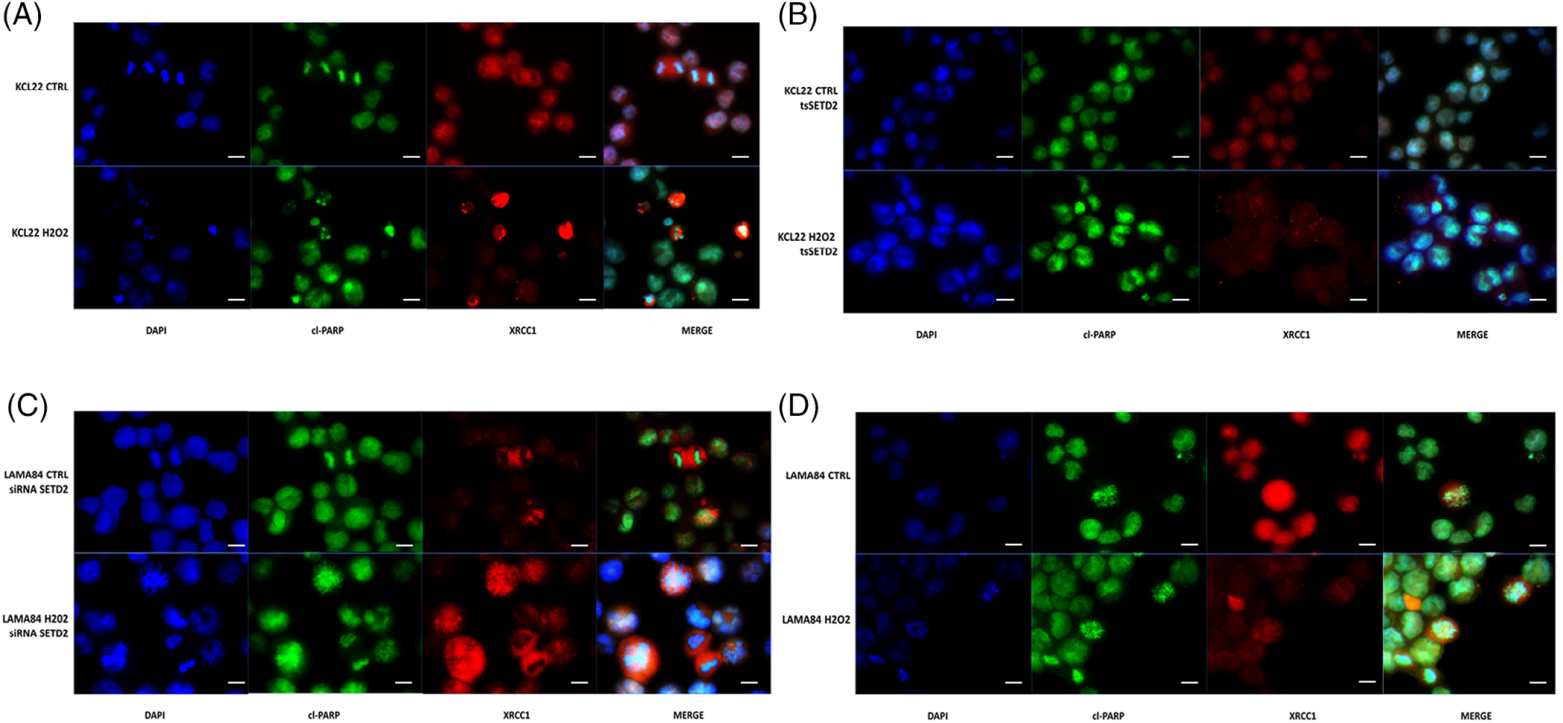
Effects of SETD2 loss of function on MMEJ activation. (A–D) Immunofluorescence analysis of cleaved‐PARP (green) and XRCC1 (red) in SETD2‐deficient and SETD2‐proficient cells after hydrogen peroxide exposure. Staining with DAPI (4′,6‐diamidino‐2‐phenylindole) indicates the nuclear localization of XRCC1 and cleaved‐PARP. (A) KCL22 (SETD2‐deficient) cells; (B) KCL22 tsSETD2 cells; (C) LAMA 84 cells silenced for SETD2; (D) LAMA 84 (SETD2‐proficient) cells. Scale bar: 100 µM.

To assess if the more error‐prone non‐homologous end joining (NHEJ) repair systems are favoured over HR at DNA DSBs, as reported in in vitro models of H3K36me3 deficiency,^35^ levels and localization of XRCC1 and cleaved PARP1 were investigated by immunofluorescence in KCL22 versus KCL22 tsSETD2, and in LAMA84 versus LAMA84 siSETD2. After H_2_O_2_ exposure, SETD2‐deficient KCL22 cells showed an increase in the expression of both XRCC1 and cleaved PARP1 proteins that co‐localized at DNA‐damage foci (Figure 6A), which was not observed in KCL22 tsSETD2 (Figure 6B). Similarly, in SETD2‐silenced LAMA84, the expression of cleaved PARP1 increased upon H_2_O_2_‐induced DNA damage, with the formation of visible DNA‐repair foci and subsequent in situ stabilization of XRCC1 (Figure 6C). In contrast, in parental LAMA84 cells expressing SETD2, H_2_O_2_ did not activate NHEJ (Figure 6D).

Experiments performed using sub‐lethal UV exposure to induce DNA damage showed superimposable results (Figure 6S).

**FIGURE 7:**
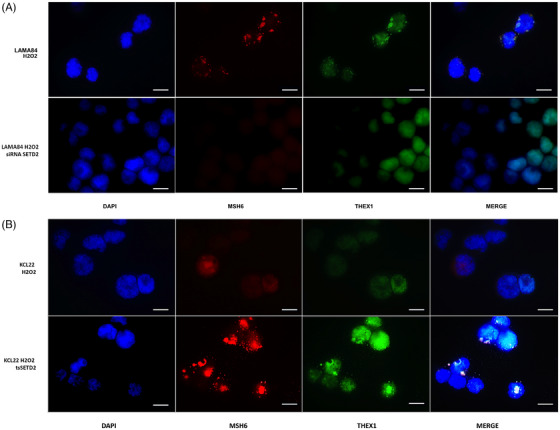
Effects of SETD2 loss of function on MMR activation. (A, B) Immunofluorescence analysis of MSH6 (red) and THEX1 (green) in SETD2‐deficient and SETD2‐proficient cells after hydrogen peroxide exposure. Staining with DAPI (4′,6‐diamidino‐2‐phenylindole) indicates the nuclear localization of MSH6 and THEX1. (A) LAMA84 parental cells versus LAMA84 cells silenced for SETD2; (B) KCL22 cells versus KCL22 SETD2‐transfected cells. Scale bar: 100 µM.

To investigate the activation of MMR in SETD2/H3K36me3‐deficient CML cells, immunofluorescence was used to check for MSH6 foci, since MSH6 is one of the partners of the hMutSα heterodimeric complex whose role is to recognize base–base mismatches or small indels and trigger MMR. Our results showed that while SETD2‐proficient cell lines (LAMA84 and KCL22tsSETD2), appear to activate MMR in response to H_2_O_2_ exposure, as suggested by MSH6 localization at discrete DNA damage foci, SETD2‐deficient cells (SETD2‐silenced LAMA84 and KCL22) were not able to initiate MMR (Figure 7A,B). Moreover, as suggested by immunofluorescence detection of THEX1 (an exonuclease involved in the induction of a rapid turnover of histone mRNA transcripts and therefore associated with the completion of DNA replication), SETD2‐proficient cell lines were able to block their replicative activity to activate DNA damage repair processes.

**FIGURE 8:**
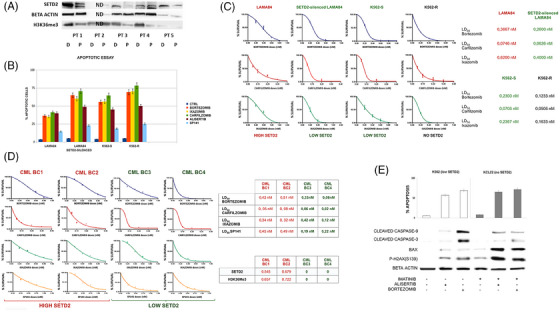
Comparison between SETD2/H3K36Me3 levels at diagnosis and progression and druggability of SETD2/H3K36me3 deficiency in cell lines and primary samples. (A) Western blot results for SETD2 protein and H3K36me3 levels in paired samples collected at diagnosis (D) and after progression (P) from 5 CML patients. Protein expression in patient 2 is not detectable (ND). One of three independent experiments is shown. (B) Flow cytometry analysis of Annexin‐V/PI positive cells. LAMA84 before and after SETD2 silencing, K562‐S and K562‐R cells were treated with 10 nM bortezomib, 40 nM ixazomib, 5 nM carfilzomib, 500 nM of alisertib and 5 µM SP141 for 24 h. Statistical analysis was performed by using dedicated software (GraphPad Prism 8.0). *p*‐values were always <.0021. (C) Dose‐dependent inhibition of LAMA84 before and after SETD2 silencing, K562‐S and K562‐R cells in 10‐day methylcellulose colony‐forming assays. Cells were treated with increasing concentrations of bortezomib, ixazomib, and carfilzomib (proteasome inhibitors). Bortezomib treatment is represented by the blue line, carfilzomib is represented by the red line, and ixazomib by the green line. (D) Dose‐dependent inhibition of the mononuclear fraction obtained by four CML BC pts showing high and low SETD2 levels in 14‐day methylcellulose colony‐forming assays. Cells were treated with increasing concentrations of bortezomib, ixazomib, carfilzomib (proteasome inhibitors), and SP141 (MDM2 inhibitor). Bortezomib treatment is represented by the blue line, carfilzomib is represented by the red line, ixazomib by the green line, and SP141 by the yellow line. (E) Flow cytometry analysis of Annexin‐V/PI positive cells. K562 (showing low SETD2 expression) and KCL22 (SETD2‐deficient) cells were treated with 500 nM of alisertib and 10 nM bortezomib for 24 h. The Student's *t*‐test was used to compare the means obtained from three independent experiments between two groups (treated cells vs. untreated). *p*‐values always <.0016. Western blot results performed in the same conditions showed marked differences in caspase 9 and 3 cleavage and Bax and phosphorylated H2AX expression levels after alisertib and bortezomib treatments. Beta‐actin was used as a loading control. One of three independent experiments is shown in all panels.

Results obtained by WB and immunofluorescence were supported by cytofluorimetric assays performed to quantify p‐H2AX (S139), RAD51, MSH6, XRCC1 expression and PARP cleavage confirming HR and MMR activation after DNA damage induction in SETD2‐proficient cells and NHEJ and Base Excision Repair activation in SETD2‐deficient cells (Figures 7S and 8S).

Taken together, these findings suggest that SETD2 loss of function may contribute to genetic instability, which is a hallmark of advanced‐phase CML.

### 3.5 SETD2/H3K36me3 deficiency observed in advanced‐phase patients appears not to be traceable back to diagnosis

To explore whether SETD2/H3K36me3 deficiency can be traced back to diagnosis, we used WB to assess SETD2 levels in matched samples collected at diagnosis and after disease progression from five CML patients. In four pairs in which proteins were evaluable, we found that SETD2 and H3K36me3 deficiency was not yet evident at diagnosis, suggesting that it must occur sometime before, or at the time of, disease progression (Figure 8A).

### 3.6 SETD2 non‐genomic loss of function in advanced‐phase CML can be therapeutically targeted

Having established that in the great majority of advanced‐phase CML patients, SETD2 loss of function is reversible, we wondered whether restoring physiological levels of H3K36me3 by pharmacological targeting of Aurora‐A kinase‐/MDM2‐/proteasome‐mediated degradation of SETD2 might represent a therapeutic strategy. We thus investigated the activity of an Aurora‐A inhibitor (alisertib, currently being evaluated in clinical trials in various neoplastic conditions including acute myeloid leukemia^43^), of SP‐141, and of two generations of proteasome inhibitors (bortezomib, carfilzomib, and ixazomib).

Apoptotic assays performed in LAMA84 cells before and after SETD2 silencing, in K562 (SETD2/H3K36me3Low), and in K562‐R cells that have lost SETD2 expression and activity, suggested that inhibition of cell proliferation associated with apoptotic activation, after proteasomal, Aurora‐A or MDM2 inhibition is strictly dependent on SETD2 expression and functional status (Figure 8B).

Clonogenic assays in LAMA84 cells before and after SETD2 silencing, in K562 and in K562‐R cells that have lost SETD2 expression and activity confirm that reduction of clonogenic potential after proteasomal inhibition is SETD2 dependent. The extent of reduction of clonogenic growth was indeed inversely correlated to SETD2 residual expression (Figure 8C).

First and second‐generation proteasome inhibitors (bortezomib, carfilzomib and ixazomib) inhibited the clonogenic potential of the mononuclear cell fraction from BC CML patients (*n* = 4) at subnanomolar concentrations, with the extent of antiblastic activity clearly anticorrelated with SETD2 expression and H3K36me3 levels: patients with lower SETD2 expression showed lower EC50 when compared with patients with higher SETD2 expression and H3K36me3 levels (Figure 8D). Similarly, clonogenic assays performed by administrating increasing doses of SP‐141 (from .25 to 1.25 µM) suggested that MDM2‐specific inhibition had more significant effects in BC‐CML patients showing low SETD2 levels and activity as compared with BC‐CML patients showing intermediate SETD2 levels and activity.

Further experiments were performed in K562 and KCL22 cell lines to verify if the reduced clonogenic potential was due to cytostatic or cytotoxic effects. Aurora‐A de‐phosphorylation by alisertib caused an increase of annexin‐V‐positive cells by activating the mitochondrial apoptotic pathway, as reflected by an increase in Bax expression and induction of the cleavage of caspase‐3 and caspase‐9. Moreover, alisertib and bortezomib as single agents at nanomolar doses (500 and 10 nM, respectively) induced a significant increase in the DNA DSB marker p‐H2AX(S139), suggesting that in a SETD2 knock‐down context, proteasomal or Aurora‐A inhibition activates the mitochondrial response to genomic instability by triggering cell death programs as DNA‐damage response (Figure 8E).

## 4 DISCUSSION

Advanced‐phase CML is characterized by a profound genetic heterogeneity.^4^ For the first time, we show evidence of a novel, nearly unifying alteration in patients with AP and BC and in patients with (multi)‐TKI‐resistant CP harbouring BCR::ABL1 KD mutations.

Given that SETD2 is the only methyltransferase able to trimethylate H3K36,^7^ and based on our previous findings in systemic mastocytosis (SM) where SETD2 loss of function leading to H3K36me3 impairment occurred much more frequently at the post‐translational rather than at the genetic/genomic or transcript level,^25^, ^26^ we opted for a WB‐based screening strategy to assess H3K36me3 as a surrogate marker of SETD2 loss of function, whichever the underlying cause. This allowed us to discover that H3K36me3 deficiency occurs in nearly all advanced‐phase CML patients, with ‘advanced‐phase’ here intended at large to include even CP patients harbouring TKI‐resistant mutations—who have actually been shown to have a ‘biologically advanced’ disease based on their gene expression signatures^44^—but not in newly diagnosed CP patients (at least not those destined to achieve optimal responses). H3K36me3 deficiency was almost always (86% of cases) paralleled by SETD2 protein deficiency. Since sequencing revealed mutations in only two patients and gene expression studies ruled out transcriptional repression or gene deletions/haploinsufficiency, we turned to interrogate translational and post‐translational mechanisms. We found that inhibition of proteasome‐mediated degradation was sufficient to re‐express a (hyper‐ubiquitinated) SETD2 protein and to restore H3K36me3, indicating that in the majority of advanced‐phase CML patients, a functional SETD2 protein is regularly translated, but undergoes aberrant turnover. This mechanism of non‐genomic loss of function, which we have recently observed also in advanced SM,^25^ let us suppose that the frequency of SETD2 loss of function in solid and hematologic malignancies might be greater than sequencing and copy number data have so far suggested. SETD2 loss by enhanced proteasomal degradation was found to be BCR::ABL1‐independent, since unlike proteasome inhibition, ABL1 kinase inhibition by TKI treatment had no effects on SETD2 protein re‐expression. In an attempt to identify the key players implicated in SETD2 loss, we incurred in MDM2 and Aurora‐A kinase. Our data point to a direct role for both Aurora‐A and MDM2 in SETD2‐enhanced proteasome‐mediated degradation in advanced‐phase CML: Aurora‐A can be hypothesized to provide the phosphorylation signals triggering MDM2‐mediated hyper‐ubiquitination of SETD2. We had previously reported that Aurora‐A is overexpressed and hyper‐activated in TKI‐resistant CML cell lines and patients.^32^ We here additionally show that expression and activation of Aurora‐A are indeed inversely correlated to SETD2 levels in CML patients. Upregulation of MDM2 in BC CML has been known for a long time,^45^ and it has recently been linked to ADAR1‐mediated A‐to‐I RNA editing of the 3′‐UTR of MDM2, enabling MDM2 to evade miRNA targeting.^46^

Several lines of evidence indicate that H3K36me3 participates in DNA damage response by directly recruiting the DNA repair machinery where needed^31^, ^40^, ^41^, ^47^ and, as a consequence, H3K36me3 deficiency has been found to be associated with low DNA repair efficiency in multiple experimental models. Firstly, H3K36me3 has been implicated in DSB repair.^31^, ^41^, ^47^

DSBs are repaired either by HR or by NHEJ. Although the cell‐cycle phase contributes to the choice between HR and NHEJ, the chromatin context in which a DSB occurs also plays an important role, since transcriptionally active, H3K36me3‐enriched chromatin has been found to be preferentially repaired by HR whereas H3K36me3‐depleted regions seem to be repaired by more error‐prone mechanisms like NHEJ^46^ or base excision repair (BER).^40^

H3K36me3 thus defines a local chromatin state that not only favours the recruitment of Rad51 to DSBs but also promotes successful HR repair.^47^ Additionally, the observation that H3K36me3 interacts in vitro and in vivo with the PWWP domain of the hMSH6 subunit of the hMutSα DNA‐damage sensor has recently sparked studies aimed at investigating the role of H3K36me3 and the effects of SETD2 loss on MMR. MMR corrects base–base mismatches and indels of simple repeated sequences arising as occasional errors during DNA replication.^48^ Being H3K36me3 physiologically enriched in the exonic regions of actively transcribed open chromatin,^49^ H3K36me3‐mediated MMR has been reported to particularly safeguard highly expressed protein‐coding genes not only during replication, by efficiently correcting mispairs in early replicating chromatin, but also during transcription, by directly or indirectly removing DNA lesions associated with a persistently open chromatin configuration.^50^ Microsatellite instability (MSI) is a common hallmark of MMR‐defective cells.^48^ However, SETD2‐depleted HeLa cells were found to display MSI to a small degree,^40^ and H3K36me3‐deficient ccRCC cell lines and patients did not show any evidence of MSI,^22^ probably because of the preferential role of H3K36me3‐mediated MMR in correcting errors and DNA damage occurring during transcription. In line with this, a series of studies performed in the past failed to detect evidence of MSI in CML, even in BC cells.^51^, ^52^, ^53^ A second readout of defective MMR is the increase in spontaneous mutation frequency.^54^ H3K36me3 depletion resulting from SETD2 knock‐out has been reported to increase the mutation frequency in actively transcribed genes but to have little influence on the mutation frequency in transcriptionally inactive regions.^50^ hMutSα recognizes a wide variety of DNA lesions including those normally processed by BER; thus it cannot be excluded that H3K3Me3 deficiency impacts on other DNA repair pathways. Accordingly, H_2_O_2_‐induced DNA lesions, which are normally substrates of BER, preferentially caused mutations in actively transcribed genes in H3K36me3‐deficient cells.^50^ Philadelphia chromosome‐positive cells per se display genetic instability, that fosters the accumulation of additional chromosomal abnormalities and point mutations in BCR::ABL1 and other genes, and that is thought to be primarily promoted by BCR::ABL1 itself. Several studies have contributed to dissecting this issue, showing that BCR::ABL1 increases endogenous DNA damage and compromises the activation and fidelity of DNA repair, inhibiting HR and MMR and stimulating error‐prone NHEJ and single‐strand annealing in response to DSBs, and BER in response to mutagenic lesions.^55^, ^56^, ^57^, ^58^, ^59^ However, our observations in SETD2 on/off CML cell line models indicate that SETD2 depletion further impacts on the proficiency of HR and MMR. Hence, it is tempting to speculate that SETD2/H3K36me3 deficiency synergizes with BCR::ABL1 to enhance genetic instability over the threshold that is necessary and sufficient to promote and/or sustain disease acceleration/progression that may ultimately lead to BC. This could provide an explanation as to why some patients develop mutations and progress despite initial response to TKI therapy, which should minimize BCR::ABL1‐dependent genetic instability.

Whether SETD2 and H3K36me3 loss precedes or follows CML progression from CP to BC has important biological and clinical implications. On one hand, it appears that newly diagnosed CP patients do not display significantly lower levels of SETD2 protein than those detectable in HD, independently of whether they will later progress to BC—although only four diagnosis/progression samples were available for paired analysis. On the other hand, TKI‐resistant CP patients harbouring BCR::ABL1 KD mutations already show evidence of SETD2 deficiency (that might be speculated to play a role in the ‘mutator’ phenotype displayed by such patients, who have been shown to have a greater likelihood of acquiring additional mutations once switched to subsequent‐line therapy^60^). Hence, it can be speculated that SETD2/H3K36me3 levels begin to decrease at some point when the patient is still in CP, before the disease eventually transforms. The hypothesis that a deficiency in SETD2 and H3K36me3 (which can be rapidly assessed using cost‐effective and straightforward methods) could serve as an early warning signal is compelling, particularly in light of the critical importance of preventing disease progression, rather than treating, disease evolution.

In conclusion, our results define in CML a pathogenetic pathway in which the aberrant expression and activation of Aurora kinase A initiates MDM2‐mediated ubiquitination of SETD2, as previously described in advanced systemic mastocytosis.^25^ This process results in increased proteasomal degradation, ultimately leading to a deficiency in H3K36Me3. Additionally, our research highlights promising therapeutic strategies for patients in advanced phases of CML, utilizing readily repurposable targeted agents (proteasome or Aurora‐A inhibitor) specifically in a context characterized by SETD2 and H3K36me3 deficiency.

Notably, in vitro and ex vivo studies have demonstrated the efficacy of proteasome inhibitors and Aurora kinase A inhibitors in patients exhibiting SETD2 loss. Accordingly, we advocate for the further exploration of these inhibitors as salvage therapies for patients with SETD2 non‐genomic loss of function who are either unresponsive to or intolerant of TKIs.

Furthermore, our observations highlight SETD2/H3K36me3 deficiency as a reversible, and druggable mechanism of genetic instability that operates independently of BCR::ABL1, potentially contributing to disease progression in CML.

## AUTHOR CONTRIBUTIONS

Simona Soverini, Gabriele Gugliotta, and Manuela Mancini designed and supervised the study and analyzed and interpreted the results. Sara De Santis, Cecilia Monaldi, Maura Rossi, Claudio Agostinelli, Manja Meggendorfer, and Torsten Haferlach performed experiments and analyzed and interpreted the results. Fausto Castagnetti, Miriam Iezza, Alessandra Iurlo, Sara Galimberti, Marco Cerrano, Isabella Capodanno, and Massimiliano Bonifacio provided patient samples and clinical data. Michele Cavo coordinated the clinical and research activities. All authors contributed to drafting the manuscript and gave final approval for submission.

## CONFLICT OF INTEREST STATEMENT

The authors declare no conflict of interest.

## Supporting information



Supporting Information

## Data Availability

All data generated or analysed during this study are included in this published article (and its supplementary information files). Methods described in this manuscript will be freely available upon request to any researcher wishing to use them for non‐commercial purposes.
