# Supplementary material for: SETD2 loss of function is a recurrent event in advanced‐phase chronic myeloid leukemia and contributes to genomic instability: SETD2 loss in Chronic Myeloid Leukemia
Source: Clin Transl Med. 2025 Apr 24;15(4):e70163. doi: 10.1002/ctm2.70163 (PMC12022228; doi:10.1002/ctm2.70163)
Supplement: Supplementary file 1 — Supporting Information [file CTM2-15-e70163-s001.docx]

**Supplementary material**

**Supplementary methods**

**Patient samples.**

Samples collected from 75 advanced-phase CML patients (AP, n=22; myeloid BC, n=37; lymphoid BC, n=16) and 9 CP patients with multi-TKI-resistant disease harboring two or more BCR::ABL1 kinase domain mutations (mut-CP) were screened for *SETD2* gene mutations, *SETD2* transcript levels, SETD2 protein expression and total H3K36me3 levels. DNA, RNA and proteins were obtained from total leukocytes using the AllPrep DNA/RNA/Protein Kit (Qiagen, Hilden, Germany). For transcript and protein levels, samples collected at diagnosis from CP pts (n=23) who achieved optimal response to therapy and samples from healthy donors (HD) were also studied for comparison. For 2 patients with myeloid BC and 2 patients with lymphoid BC, matched samples before and after progression were available. For 2 patients in myeloid BC and 2 patients in lymphoid BC, cryopreserved cells were also available for drug studies. Sample collection was approved by the Institutional Review Boards of the S. Orsola-Malpighi Hospital (protocol 112/2014/U/Tess) and of the participating institutions.

**RNA interference.**

A transient short interfering RNA approach was used to silence *SETD2* in LAMA84 cells and to silence *MDM2* and *AURKA* in K562 cells. Briefly, cells were plated at a density of 10^5^ cells/mL in a 6-well plate. After 24 hours (h), cells were transfected using 100 pM siRNA positive control duplex, or 100 pM negative control duplex or 100 pM of the gene-specific siRNAs (Integrated DNA Technologies, Coraville, IA, USA), mixed in 500 μL of serum-free medium and 7.5 μL of TransIT-siQUEST Reagent (Mirus, Madison, WI, USA). The siRNA duplex sequences selected (among three different sequences tested) to silence S*ETD2*, *MDM2* and *AURKA* were 5’-AAGAAUAAAUCUCAUCGAGAUAUTA-3’, 5’-CUUUACAUGUGCAAAGAAGCUAAAG.3’, and 5’-GACAGGAACAUGCUACUGAAGUUTA-3’, respectively. Twenty-four hours post-transfection, RNA was isolated and target transcript levels were measured by RT-qPCR using the following TaqMan gene expression assays (ThermoFisher Scientific), *SETD2*: Hs01014784_m1; *MDM2*: Hs01066930_m1; *AURKA*: Hs01582072_m1. Relative expression was normalized to *GUSB* mRNA (Hs00939627_m1) and the negative control was used as baseline (100%); 24, 48 and 72h post-transfection, protein expression was assessed by Western blotting (Figure S1 A).

**Co-immunoprecipitation/immunoblotting and Western blot analyses.**

WB was performed using 40 μg of whole cell lysates, separated on 10% Tris-glycine gels, transferred, and blotted. Immunoprecipitation (IP) was performed using 250 µg of whole cell lysates in the presence of Cyanogen bromide (CNBr)-activated sepharose 4B (GE Healthcare, Chicago, IL, USA) conjugated with an anti-SETD2 antibody (Genetex, Irvine, CA, USA). IP products were resolved by SDS-PAGE, blotted and labeled with primary and secondary antibodies. Immunoreactive proteins were visualized by probing with horseradish peroxidase-conjugated secondary antibodies and then by enhanced chemiluminescence (ECL; Thermo Fisher Scientific). Blots were scanned by using a Chemidoc XRS+ (Bio Rad Laboratories, Hercules, CA, USA), In patient samples, band intensities for the SETD2 protein were quantified with the ImageJ software. Signal intensities in single blots obtained from three individual experiments were averaged, normalized to those of the loading controls and expressed as a fraction of the intensities, set to 1, obtained in a pool of 10 healthy donors of various ages used as a control.

**Immunofluorescence.** Cells set on poly-L-lysine-coated glass slides were fixed with 3.7% paraformaldehyde in phosphate-buffered saline (PBS) for 10 minutes (min) at 37°C, washed three times with 0.1M glycine in PBS, permeabilized in 70% ice-cold ethanol for 2 min at -20° C and incubated overnight at 4°C with primary anti phospho-H2AX S(139) –(diluted 1/200 in BSA1% in PBS according to the manufacturer's instructions), anti-RAD51 antibodies (diluted 1/200 in BSA1% in PBS according to the manufacturer's instructions), or with anti-MSH6 (diluted 1/50 in BSA1% in PBS according to the manufacturer's instructions) and anti-THEX1 (diluted 1/50 in BSA1% in PBS according to the manufacturer's instructions) antibodies, or with anti-XRCC1 (diluted 1/100 in BSA1% in PBS according to the manufacturer's instructions) and anti-cleaved-PARP1 (diluted 1/400 in BSA1% in PBS according to the manufacturer's instructions) antibodies. Slides were then incubated with a secondary FITC- or Alexa Fluor 568-conjugated anti-rabbit antibody (both diluted 1/1000 in BSA1% in PBS according to the manufacturer's instructions) for 1h at room temperature, and a subsequent labelling with 6-diamidino-2-phenylindole (DAPI diluted 0.1 µg/ml in PBS was incubated for 10 minutes at room temperature in the dark) was used to stain the nuclear compartment. All antibodies were purchased from Cell Signaling Technology. Analyses were performed using an Axiovert 40 CFL microscope (Carl Zeiss S.p.A., Reggio Emilia, Italy). Images were acquired with a 100X objective.

**Drug treatments.** Imatinib and nilotinib (Selleckchem, Houston, TX, USA) 1 µM and 100 nM, respectively, were used to inhibit BCR::ABL1 tyrosine kinase activity. Bortezomib (first generation proteasome inhibitor), ixazomib and carfilzomib (second generation proteasome inhibitors), all purchased from Selleckchem, were used to investigate the effects of proteasomal inhibition on SETD2 expression and activity. SP141 (Selleckchem) was used to assess the effects of MDM2 inhibition on SETD2, and alisertib (Selleckchem) was used to investigate the effects of selective Aurora kinase A inhibition on SETD2. UV chronic exposure (30 min three days a week for 14 weeks) and hydrogen peroxide single exposure (1mM for 30 and 60 min) were performed to test DNA damage repair differential ability of SETD2 proficient and deficient cell lines. The biological consequences on apoptotic cell death activation of the aforementioned drugs were evaluated after 24h treatment with each compound (10 nM bortezomib, 40 nM ixazomib, 5 nM carfilzomib, 5 µM SP141, 100nM of alisertib). Apoptotic cell death was assessed by measuring the uptake of ﬂuoresceinated Annexin V and propidium iodide (PI) (F. Hoffmann-La Roche Ltd., Basel, Switzerland) according to manufacturer’s instructions. A FACSCantoII ﬂow cytometer (Beckton Dickinson, Franklin Lakes, NJ, USA) set at 488nm excitation and 530nm wavelength bandpass ﬁlter for ﬂuorescein detection or 580nm for PI detection, and a dedicated software (DIVA software, Beckton Dickinson) were used.

Drug efficacy was evaluated by clonogenic assays. In CML cell lines and mononuclear cell fractions from primary samples, the reduction of colony number generated in StemMAC HSC-CFU Media (Miltenyi Biotec, Bergisch Gladbach, Germany) in the presence of increasing doses of bortezomib, ixazomib, carfilzomib, was assessed after 10 and 14 days of incubation at 37°C in a fully humidified atmosphere and 5% CO_2_, respectively. Nonlinear regression analyses (GraphPad Prism; GraphPad Software Inc.) were used to calculate the effective dose (ED50) of the different drugs.

**Cytofluorimetric assays.** Cytofluorimetric assessment of protein expression and post-translational modifications was performed to obtain a quantitative evaluation of homologous recombination (HR) repair, mismatch repair (MMR), base excision repair (BER) and non-homologous end-joining (NHEJ) repair activation after DNA damage induction. Briefly, KCL22, KCL22 tsSETD2, LAMA84 parental and silenced for SETD2 cell lines before and after H_2_O_2_ or UV administration were pelleted by centrifugation and supernatant was removed. 10^6^ cells were then washed and fixed in 4% formaldehyde for 15 minutes at room temperature (20-25°C). Permeabilization was carried out by adding -20° C 100% methanol slowly to pre-chilled cells, while gently vortexing, to a final concentration of 90% methanol. Cells were then washed by centrifugation to remove methanol and incubated in 100 µl of diluted primary antibodies (anti-p-H2AX(S139), anti-RAD51, anti-MSH6, anti-XRCC1 and anti-cleaved-PARP1), prepared in an antibody dilution buffer (BSA 1% in PBS) at the recommended dilution for 1 hour at room temperature. All antibodies were purchased from Cell Signaling Technology, Danvers, MA, USA. Cells were resuspended in 100 µl of diluted fluorochrome-conjugated secondary antibody (prepared in antibody dilution buffer at the recommended dilution) and incubated for 30 minutes at room temperature. Finally, cells were washed, resuspended in 500 µl of PBS and aquired by using a CytoFLEX analytical flow cytometer (Beckman Coulter, Brea, CA, USA).

**Supplementary data**


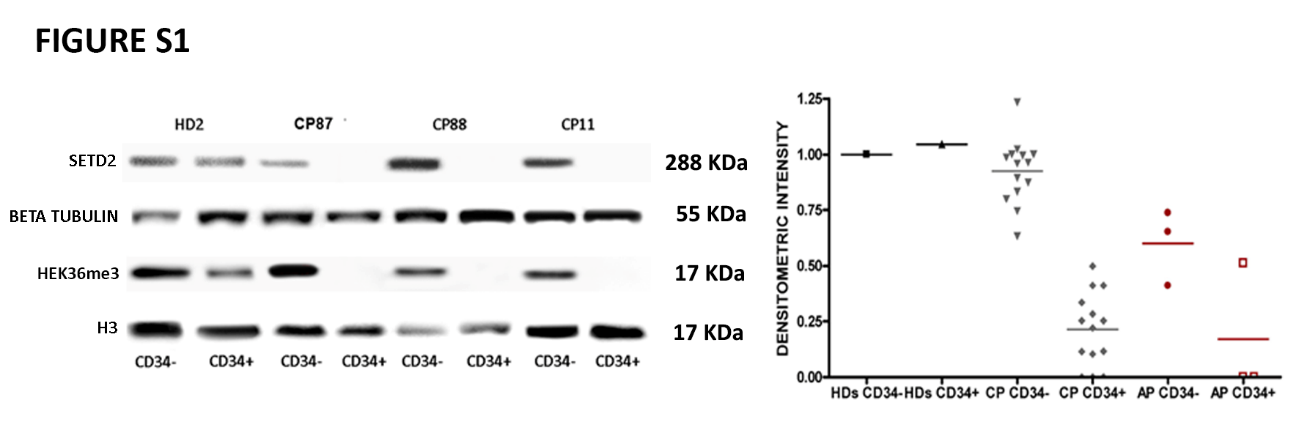


**Figure 1S: SETD2 deficiency in CD34+ compartment.** Representative Western blot results for SETD2 protein and H3K36me3 levels in CD34+ vs CD34- cells from CML patients as compared to a pool of healthy donors. **B** Scatter plot of SETD2 levels estimated by densitometric analysis of Western blots. The median is indicated for each group. SETD2 signal intensities were normalized to those of beta-tubulin and averaged. Normalized SETD2 levels calculated in CML patients were then expressed in comparison to normalized SETD2 levels detected in a pool of healthy donors, conventionally set to 1. The Student's t test was used to compare the means between two groups (CP CD34+ vs HDs CD34+ P value<0.0001; CP CD34+ vs CP CD34- P value<0.0001; AP CD34+ vs HDs CD34+ P value<0.0001; AP CD34+ vs AP CD34- P value<0.001). Abbreviations: HD, healthy donor; CP, chronic phase; AP, advanced-phase.


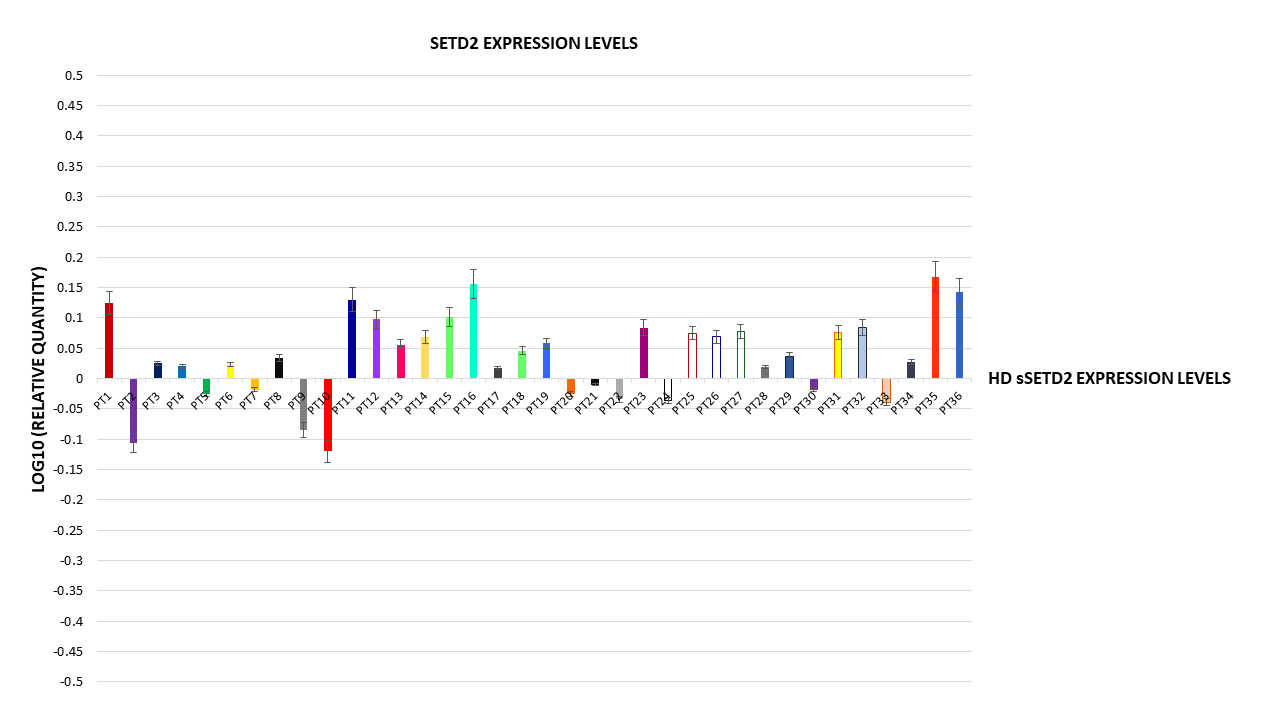


Figure 2S: Representative SETD2 mRNA transcript levels in 36 out of the 84 samples. SETD2 expression levels were normalized to GUSB (endogenous control) expression levels. and quantified using the Comparative Ct method, using a pool of 10 healthy donors of various ages as calibrator.

**
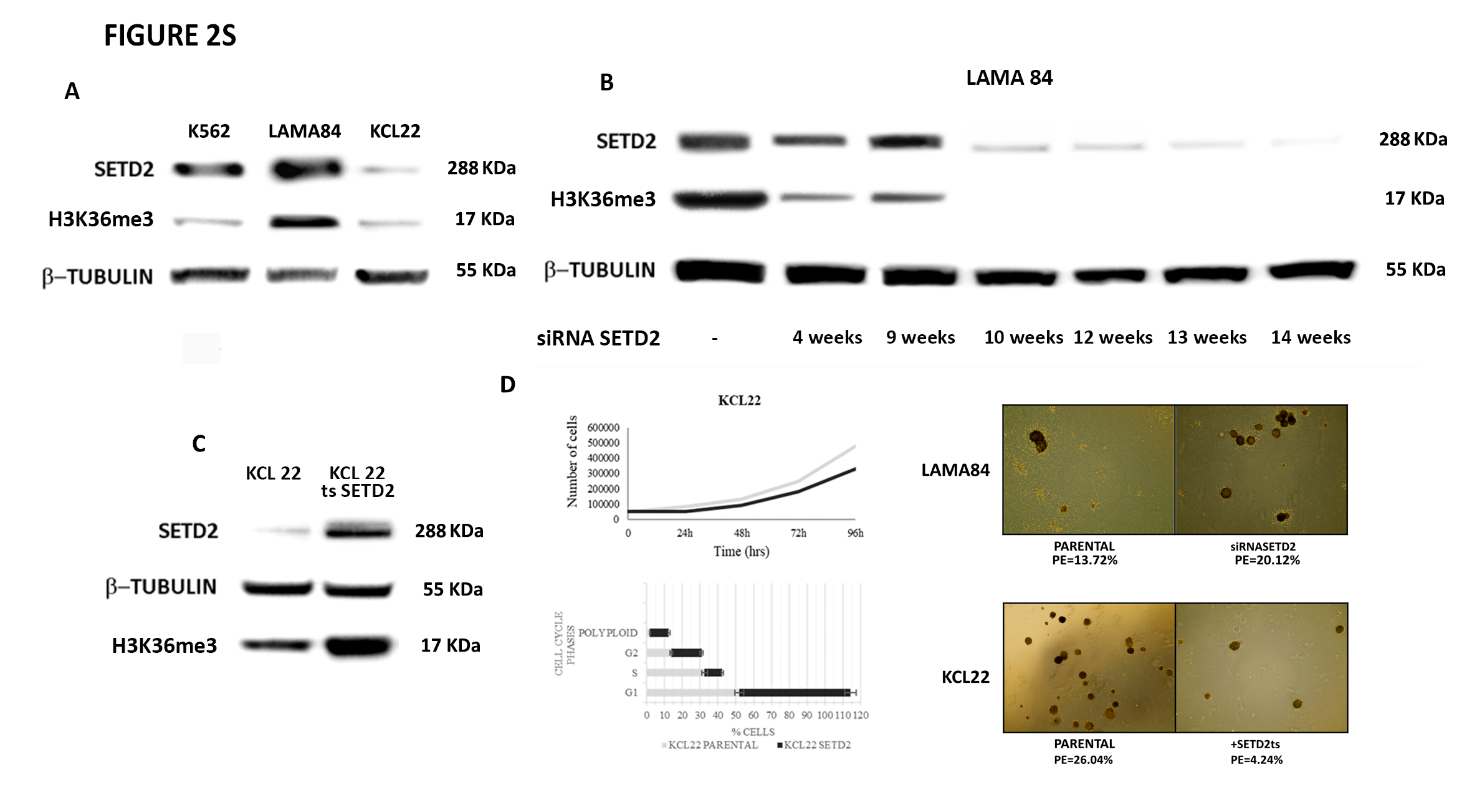
**

**Figure 3S: Western blotting experiments performed to assess SETD2 expression and activity in: A** parental K562, LAMA84 and KCL22 cell lines, **B** LAMA84 silenced for SETD2 expression for fourteen weeks. SETD2 expression and function were checked at different timepoints. **C** KCL22 cells subjected to nucleofection using the Lonza Nucleofector 2b Unit to obtain SETD2 forced expression. SETD2 expression and function were tested after 72h. **D** After forced SETD2 expression in KCL22 cells, an increase in doubling time, an accumulation of cells in G1 and G2 phases and a reduction in clonogenic capacity can be observed.


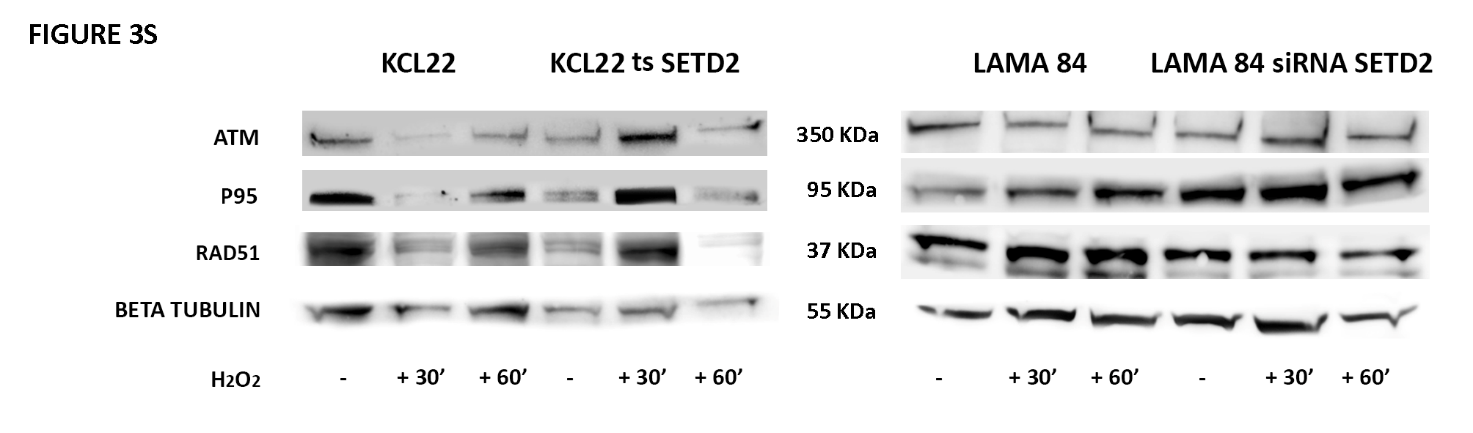


**Figure 4S: SETD2 impacts on ATM, p95 and Rad51 expression after induction of DNA damage.** Western blotting experiments showing that SETD2 deficiency in KCL22 (and LAMA84 siSETD2 cells) impairs ATM signalling pathway; in contrast, SETD2 forced re-expression in KCL22 cells restores cell’s ability to induce ATM and p95 expression, associated with an increase in Rad51 expression, after incubation with 1 mM hydrogen peroxide for 30 min. Beta tubulin was used as loading control. It has to be noted that in KCL22 tsSETD2, after 60 min-incubation with hydrogen peroxide, cells underwent massive apoptotic cell death, thus protein degradation may be observed.


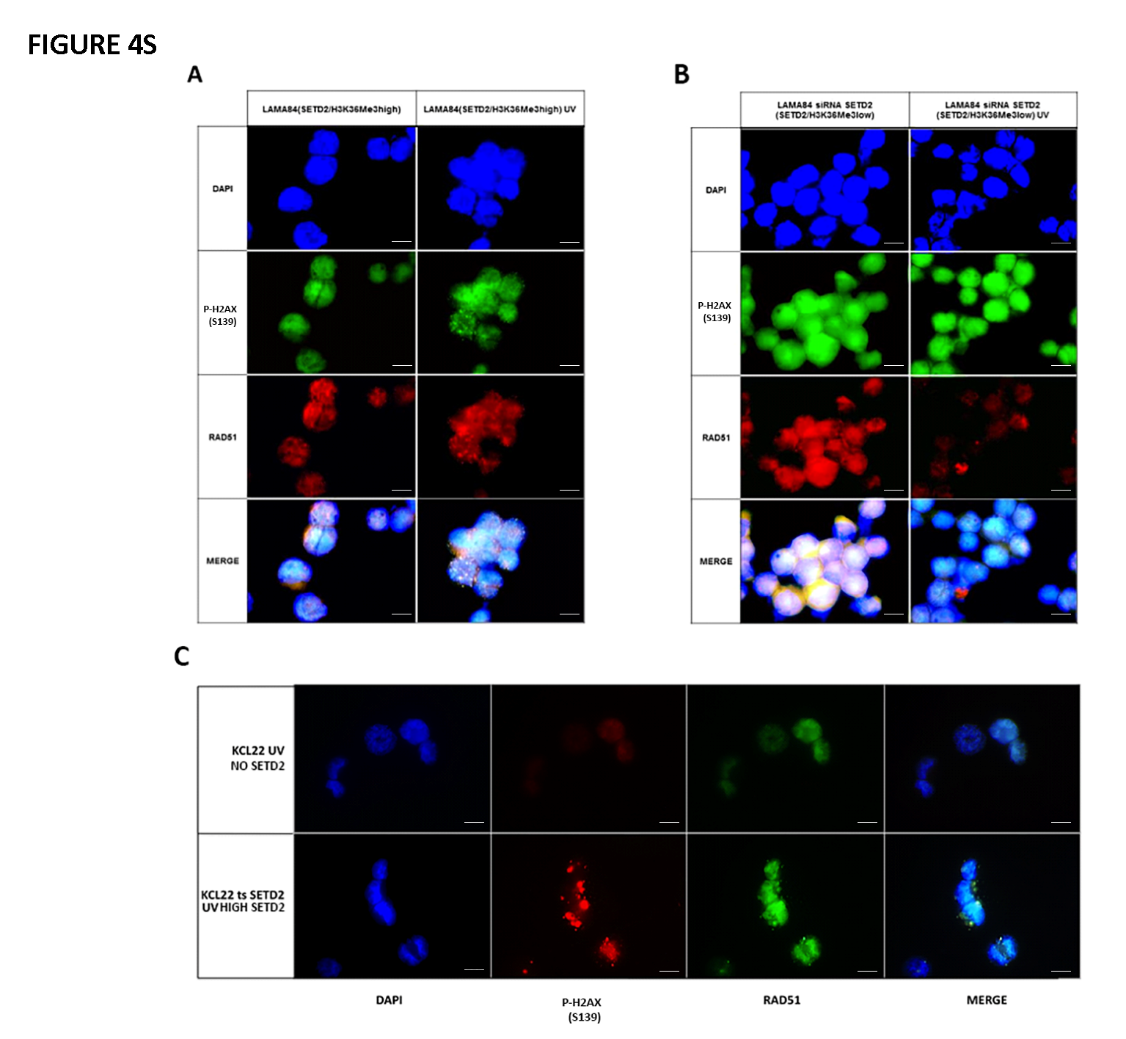


**Figure 5S: Effects of SETD2 deficiency on HR activation.** Panels A, B and C show immunofluorescence analysis of phosphorylated histone 2A.X (P-H2AX(S139), green in panel A and B and red in panel C) and Rad51 (red in panel A and B and green in panel C) in SETD2 proficient-LAMA 84 cells (**A**) compared to LAMA 84 siRNA SETD2 cells (**B**), and in KCL22 SETD2-deficient cells (**C**) compared to KCL22 tsSETD2 cells (**D**) after sub-lethal DNA damage induction by UV exposure. Staining with DAPI (4’,6-Diamidino-2-Phenylindole) indicates the nuclear localization of P-H2AX(S139) and Rad51. Scale bar: 100 µM.


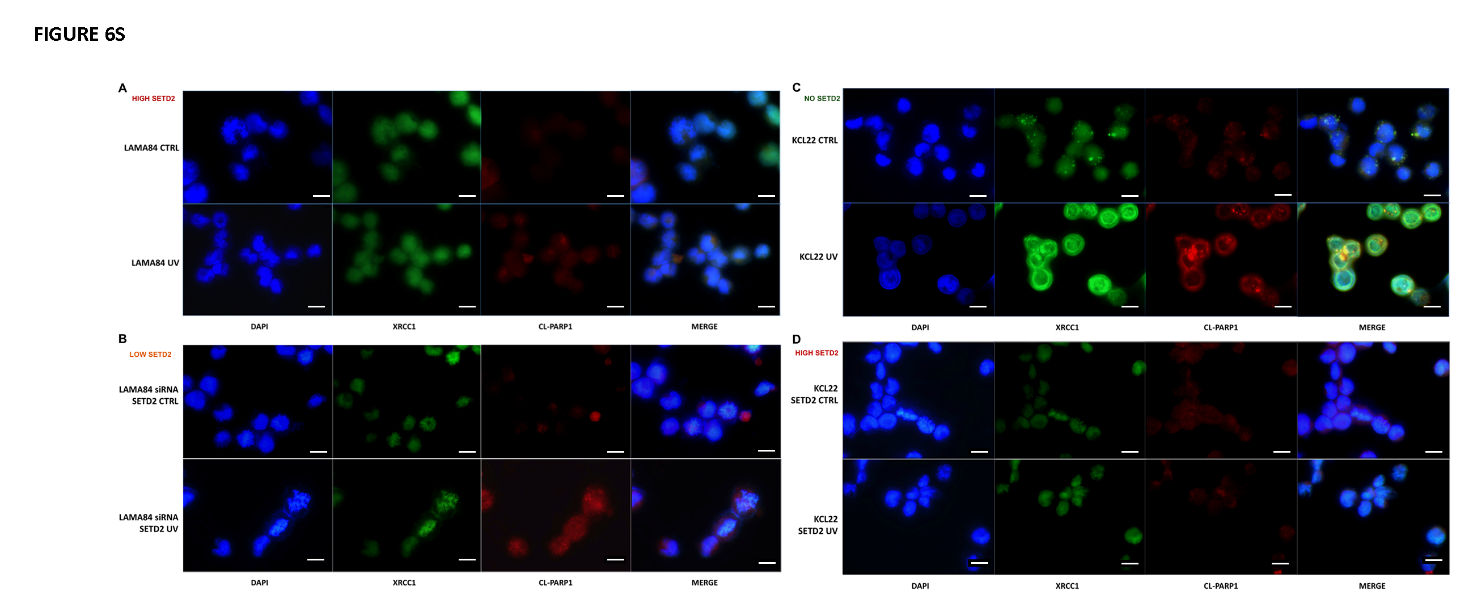


**Figure 6S:** **Effects of SETD2 deficiency on the activation of micro-homology mediated end-joining (MMEJ).** Panels A, B, C and D show immunofluorescence analysis of XRCC1 (green) and cleaved-PARP (red) in SETD2-proficient LAMA 84 cells (**A**) compared to LAMA 84 cells silenced for SETD2 (**B**) and in SETD2-deficient KCL22 cells (**C**) compared to KCL 22 tsSETD2 cells (**D**) before and after sub-lethal DNA damage induction by UV-exposure. Staining with DAPI (4’,6-Diamidino-2-Phenylindole) indicates the nuclear localization of XRCC1 and cleaved-PARP. Scale bar: 100 µM.


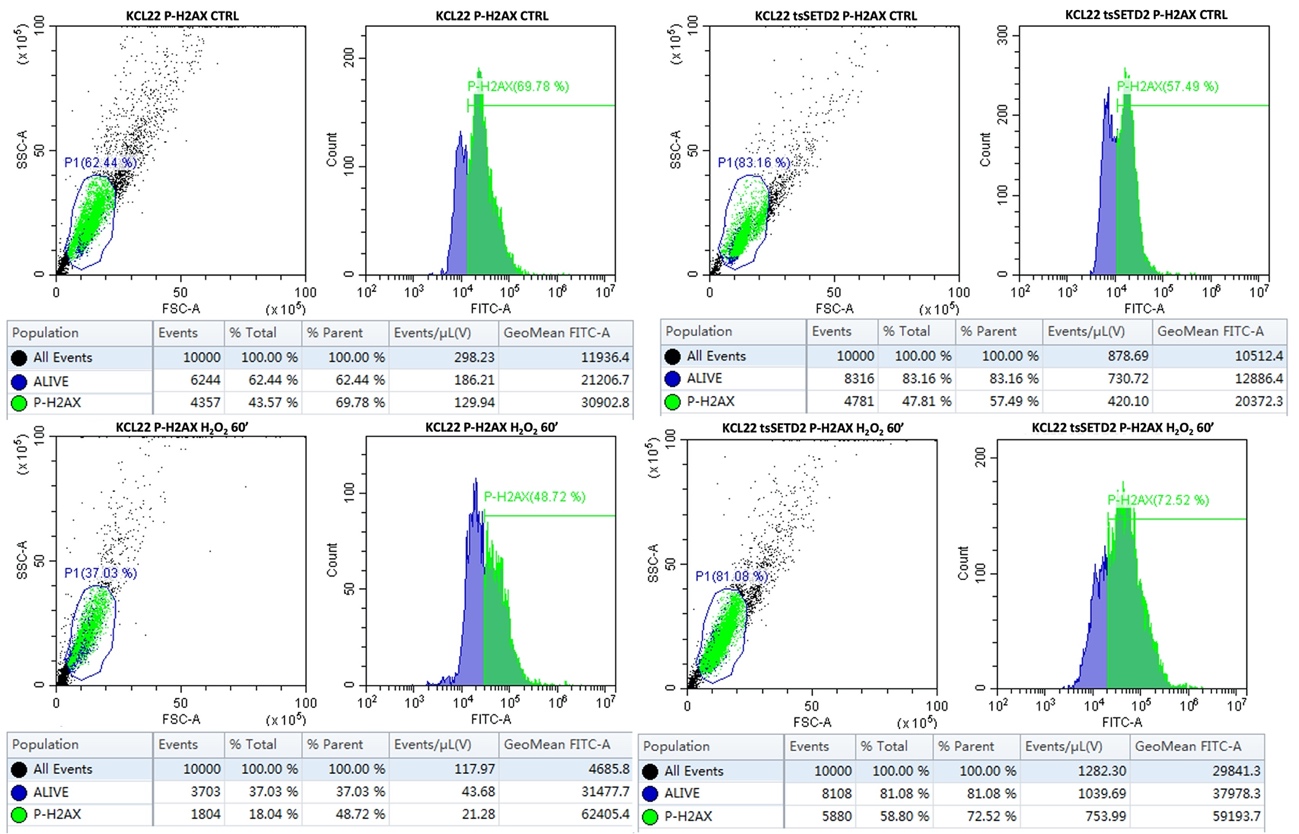


**Figure 7S: Density and histogram plots obtained by using CytExpert 2.4 software showing H2AX phosphorylation status before and after DNA damage in SETD2 deficient and proficient cells.** A gate for alive cells was established and only gated cells were considered in p-H2AX(S139) positive expression analysis in KCL22 (left panels) vs KCL22 tsSETD2 (right panels) cell lines before and after H_2_O_2_ 1 mM for 60’.

**
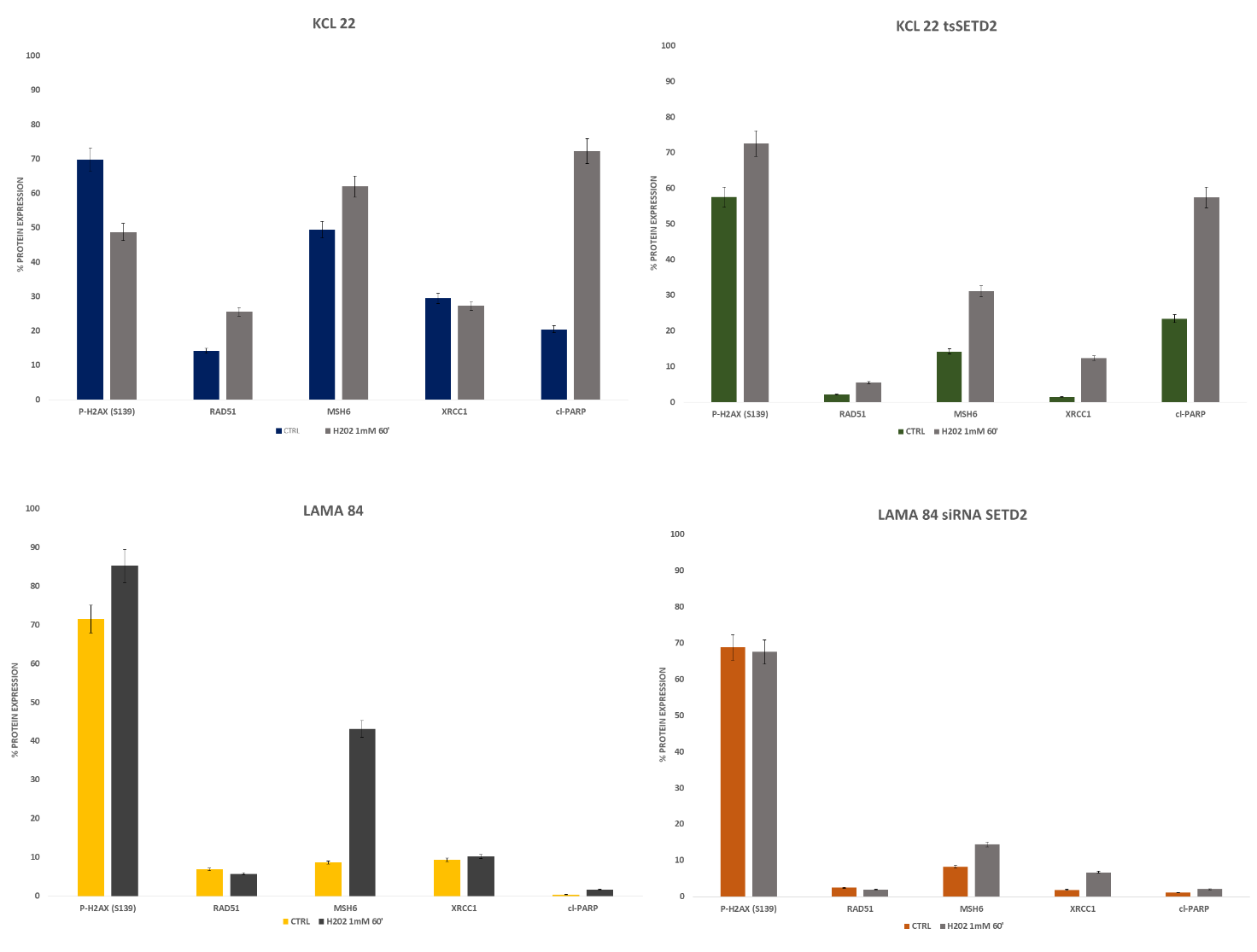
**

**Figure 8S: Cytofluorimetric assessment of HR, MMR, BER and NHEJ repair in SETD2 deficient and proficient cell lines.** A bar graph represents protein expression values: p-H2AX (S139), RAD51, MSH6, XRCC1 and cl-PARP expression in KCL22 (in steady state conditions: blue and after H_2_O_2_ damage for 60’: gray), KCL22 tsSETD2, LAMA84 and LAMA84 siRNA SETD2 cell lines evaluated in steady state condition and after H_2_O_2_ damage for 60’. On the left column each control, and on the right (grey) each damaged line. Results are a mean of three independent experiments.
